# Rapid, high efficiency isolation of pancreatic ß-cells

**DOI:** 10.1038/srep13681

**Published:** 2015-09-02

**Authors:** Susan M. Clardy, James F. Mohan, Claudio Vinegoni, Edmund J. Keliher, Yoshiko Iwamoto, Christophe Benoist, Diane Mathis, Ralph Weissleder

**Affiliations:** 1 Center for Systems Biology, Massachusetts General Hospital, Harvard Medical School, Boston, Massachusetts; 2 Division of Immunology, Department of Microbiology and Immunobiology, Harvard Medical School, Boston, Massachusetts; 3 Department of Systems Biology, Harvard Medical School, Boston, Massachusetts; 4 Evergrande Center for Immunologic Diseases, Harvard Medical School and Brigham and Women’s Hospital, Boston, Massachusetts.

## Abstract

The ability to isolate pure pancreatic ß-cells would greatly aid multiple areas of diabetes research. We developed a fluorescent exendin-4-like neopeptide conjugate for the rapid purification and isolation of functional mouse pancreatic β-cells. By targeting the glucagon-like peptide-1 receptor with the fluorescent conjugate, β-cells could be quickly isolated by flow cytometry and were >99% insulin positive. These studies were confirmed by immunostaining, microscopy and gene expression profiling on isolated cells. Gene expression profiling studies of cytofluorometrically sorted β-cells from 4 and 12 week old NOD mice provided new insights into the genetic programs at play of different stages of type-1 diabetes development. The described isolation method should have broad applicability to the β-cell field.

The development of novel therapeutics for the treatment and management of diabetes, requires a good understanding of the central component of the disease, the ß-cell. Isolation of pancreatic ß-cells is an essential procedure to study the cellular, molecular and functional aspects of these cells. Working with pure ß-cells would allow for a variety of research opportunities for type-1 and type-2 diabetes including understanding how ß-cells respond to immunotherapy, analysis of gene expression and ß-cells response to novel therapeutic regimens. However, the mosaic organization and heterogeneity of the islet has limited the isolation and characterization of the individual endocrine cell type.

Islet subpopulations are easily distinguishable by intracellular staining of their respective hormones; however the required fixation and permeabilization negates further downstream analysis. A variety of methods have been proposed in recent years to overcome the fixation and permeabilization requirement. However, although widely used, current methods for ß-cell isolation are generally complex, costly and/or do not clearly separate ß-cells from other cells. The three most common procedures rely on either i) the use of antibodies, that are not generally available, to indirectly isolate ß-cells by negative selection by staining antigens on other cells (alpha and non-endocrine)^1^, ii) the use of zinc-chelating dyes such as Newport Green^2^, which are nonspecific^3^ and do not separate ß-cells clearly from other pancreatic cells^4^ or iii) the higher level of autofluorescence naturally present in normal murine β-cells^5^. The latter method is based on the observation that murine ß-cells have a high content of flavin adenine dinucleotide (FAD). While sorting by autofluorescence is the most accepted purification method, the robustness of this approach may fluctuate in certain experimental settings, and results obtained from this method may be difficult to interpret due to the unknown overall purity of the ß-cell population^6^.

Pancreatic ß-cells highly express the cell surface glucagon-like peptide-1 receptor (GLP-1R) which has been targeted by fluorescent exendin-4 conjugates to selectively visualize ß-cells in intact islets *in vivo*^7^ and *in vitro*^8^. Exendin-4 (E4), a high affinity GLP-1R agonist, has been shown to accommodate some fluorescent modifications while maintaining GLP-1R selectivity^7^,^8^. We hypothesized that a GLP-1R agonist could be optimized for ß-cell isolation by identifying ideal reagents and conditions. Here we describe our results on one optimized method identifying BODIPY TMR-X labeled exendin-4-like neopeptide (EP12-BTMR-X) as an ideal affinity reagent for flow sorting and analysis of live mouse ß-cells. We then show the particular usefulness of this probe by analyzing microarray transcriptional profiles of isolated ß-cells in a mouse model of type-1 diabetes.

## Results

**Figure 1:**
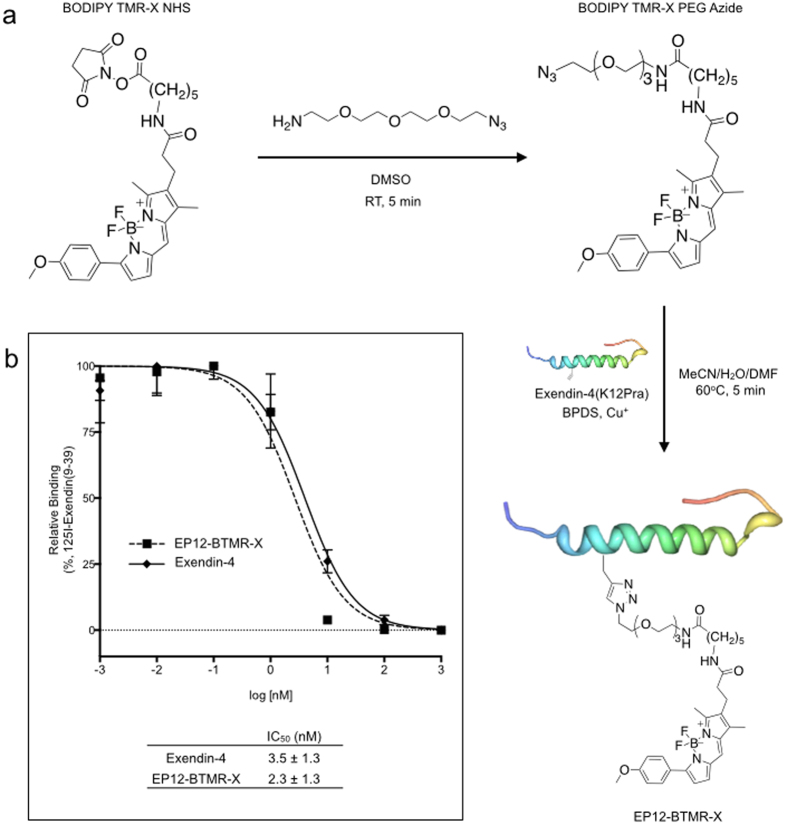
The synthetic scheme and characterization of EP12-BTMR-X. (**a**) The synthesis of EP12-BTMR-X is a simple two step reaction, which utilizes a microwave reactor to complete the reaction in 5 minutes. (**b**) The addition of BODIPY TMR-X does not significantly disrupt the high binding affinity of exendin-4 for the glucagon-like-1 receptor as demonstrated by an ^125^I-exendin (9–39) competitive binding study.

Based on the known structure-activity relationships for E4-like neopeptides^8^, amino acid position 12 was chosen for selective fluorochrome attachment to ensure GLP-1R affinity was maintained. To facilitate specific conjugation, we designed an E4-like neopeptide with propargylglycine (Pra) substituted for the lysine residue at the 12 position (EP12). EP12-BTMR-X was synthesized utilizing microwave-assisted copper catalyzed azide-alkyne Huisgen cycloaddition (Fig. 1A)^9^ and was purified by high-performance liquid chromatography. The synthesis and purification produced a ~10% yield resulting in sufficient product for over 200 individual mouse ß-cell isolations, when starting with 0.5 mg EP12 (based on 1 mL of 50 nM EP12-BTMR-X per condition). Receptor binding studies with ^125^I-Exendin (9–39) confirmed the peptide conjugate maintains high-affinity binding compared to E4 (IC_50_ = 2.3 ± 1.3 (EP12-BTMR-X) and 3.5 ± 1.3 nM (E4), Fig. 1B).

**Figure 2:**
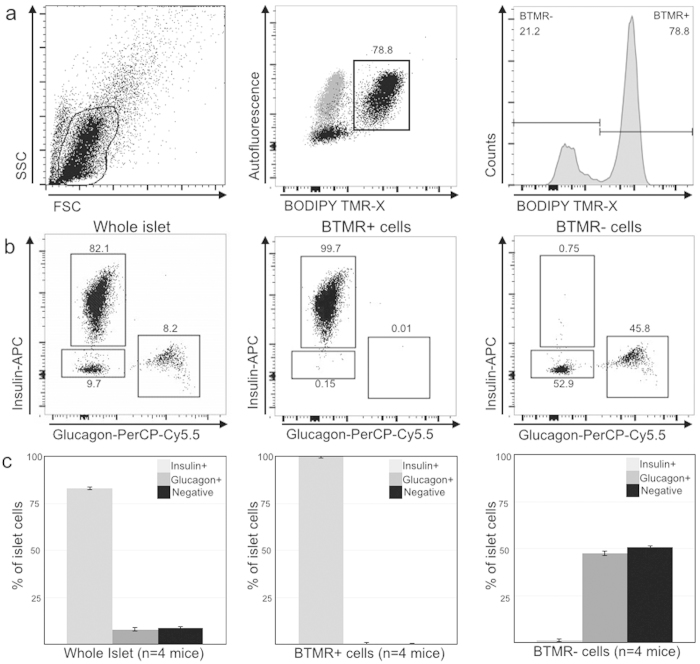
Identification of EP12-BTMR-X positive cell population. (**a**) Left. Representative flow cytometric data and gating strategy for whole islet population. Center. Cell population (grey) shift upon addition of EP12-BTMR-X (black). Right. Flow cytometric histogram of the entire cell population after EP12-BTMR-X addition. (**b**) Insulin and glucagon populations in whole islets (left) vs. EP12-BTMR-X positive (BTMR+) (center) and EP12-BTMR-X negative (BTMR-) cells (right). (**c**) Average percent insulin/glucagon populations for 4 independent islet preps.

To characterize the staining profile of this probe, freshly harvested mouse islets were incubated with EP12-BTMR-X or a BODIPY TMR-X scrambled peptide control (0.5 mL, 25 μmole, 50 nM) in standard growth media. Staining of the ß-cells with EP12-BTMR-X, within the intact islet, was completed within 30 min at 37 °C. Following staining, islet dispersion was achieved by trypsin treatment and single cells were sorted cytofluorometrically. Due to the rapid internalization and recycling of GLP-1R (~5 mins when stimulated by E4^10^), the probe was continuously internalized during the 30 min incubation, resulting in an intense fluorescent signal and an easily identified cell population in flow cytometry (Fig. 2A). A typical procedure resulted in the isolation of ~10,000 beta cells per mouse (100–150 islets). The viability of the isolated beta cells (~90%) was determined by flow cytometer analysis with DAPI staining and the ability to isolate intact RNA from sorted cells for further microarray analysis. Staining of islets was not seen with the BODIPY TMR-X scrambled peptide control, demonstrating the importance of the E4 peptide sequence for GLP-1R mediated uptake(see Supplemental Fig. 1).

Several experimental conditions (temperature, time, etc.) were explored to determine the described optimal staining condition. Staining efficiency decreased 2-fold when performed at 4 °C, likely due to the inability of GLP-1R internalization. Similar staining efficiency was obtained with overnight incubation with the probe, suggesting stability of the probe. However, we would like to stress that practically, one would add the probe and perform the isolation rather then waiting. Trypsin dispersion of the islet prior to staining with EP12-BTMR-X was also performed and resulted in similar results, however we chose to stain the intact islet to limit the time that β-cells are not in close contact with each other to reduce artifacts that may confound downstream results, such as altering RNA expression profile.

We next sought to identify the EP12-BTMR-X stained islet population with multi-parametric flow cytometry based on co-staining for islet hormones and compared our method to the commonly applied practice of identifying mouse β-cell based on intrinsic autofluorescent properties (Fig. 2)^5^. First, looking at the autofluorescence profile versus probe staining, in dispersed islet preparation (Fig. 2A, grey) the addition of EP12-BTMR-X resulted in a significant fluorescent shift (Fig. 2A, black) of the autofluorescent population. After incubation with EP12-BTMR-X, cells were fixed and permeabilized to allow for co-staining of anti-hormones antibodies. Analysis of dispersed islet cells (Fig. 2B) stained for insulin and glucagon resulted in a typical ~80/10/10 percent (beta/alpha/other cell type) distribution in the islet. Gating on EP12-BTMR-X positive (BTMR+) cells, we found 99.7% of the probe positive cells were also insulin positive. When gating on the EP12-BTMR-X negative (BTMR-) cells, we found the majority of the cells were glucagon positive insulin negative (45.8%) or negative for both insulin and glucagon (52.9%). We confirmed the BTMR+ cells were reproducibly insulin positive ß-cells but not the glucagon positive alpha cells (Fig. 2C).

**Figure 3:**
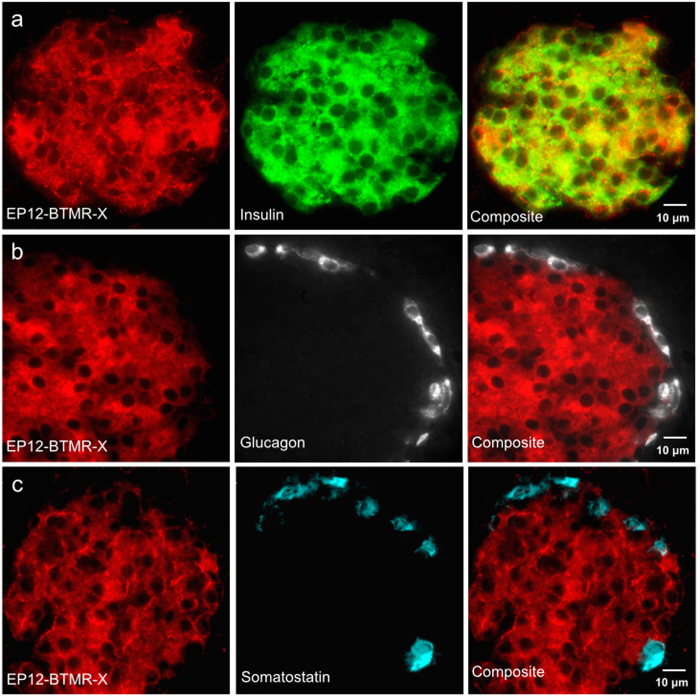
Immunostaining of ex vivo mouse pancreas for hormones insulin, glucagon and somatostatin demonstrate the specificity of EP12-BTMR-X for insulin positive beta cells. (**a**) EP12-BTMR-X (red) accumulates in insulin positive (green) cells. (**b**) EP12-BTMR-X (red) does not accumulate in glucagon positive (gray) alpha cells or (**c**) somatostatin positive (cyan) delta cells.

Probe specificity was further demonstrated with immunofluorescence microscopy on isolated mouse pancreas. Figure 3 shows fluorescent EP12-BTMR-X was localized only in insulin positive cells (Fig. 3A) or c-peptide positive (Supplemental Fig. 2) and was absent in peripheral, glucagon positive alpha cells (Fig. 3B) and somatostatin positive delta cells (Fig. 3C).

**Figure 4:**
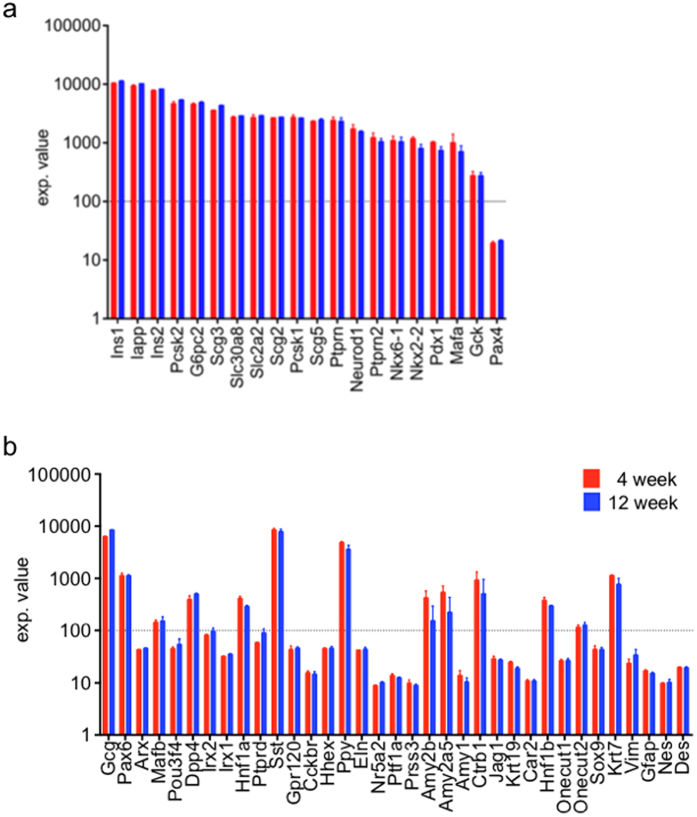
Gene expression profiling of β-cells isolated from NOD mice. BTMR + cells sorted from 4 and 12 week old NOD mice were analyzed by microarray-based transcriptional profiling. Transcript levels of selected genes preferentially expressed in either (**a**) β-cells or (**b**) other cell types.

To determine the molecular make-up of the EP12-BTMR-X positive cells, we analyzed the gene expression profiles of cytofluorometrically sorted cells from NOD mice by microarray analysis. We first investigated transcript levels of genes reported to be preferentially expressed in β-cells. Profiling revealed robust expression of canonical β-cell genes involved in insulin granule processing and secretion, such as *Pcsk2* and *Slc30a8*, as well as traditional transcription factors associated with β-cell development such as *Nkx6-1* and *Pdx1*(Fig. 4A). To ascertain the purity of our β-cell isolation we examined the expression levels of genes reported to be preferentially expressed in other pancreatic cell lineages including alpha, delta, acinar, ductal and stellate cells (Fig. 4B). Expression levels for the majority of these genes fell below the cutoff level set for reliable detection, supporting the notion that EP12-BTMR-X specifically labels β-cells. Interestingly, we also detected strong transcriptional signals in ß-cells for other endocrine hormones, including glucagon (*Gcg*), somatostatin (*Sst*) and pancreatic polypeptide (*Ppy*), an observation that is in line with previous studies that have shown β-cells to co-express multiple endocrine hormones at the RNA level^11^.

**Figure 5:**
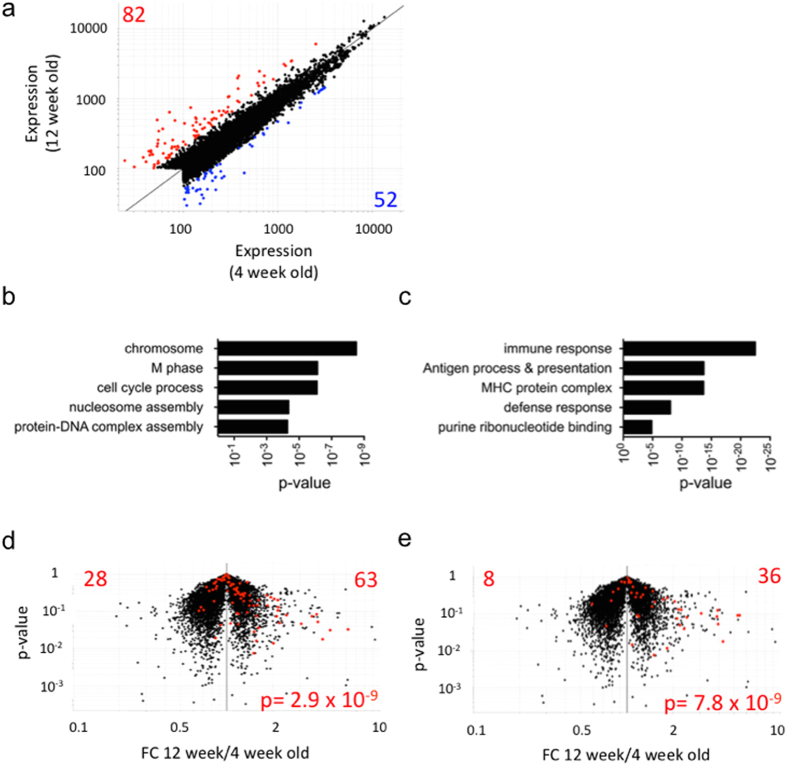
Gene expression profiling of ß-cells at different ages and stages of diabetes development. (**a**) Transcriptional profiling data depicting genes that were increased >2 fold in ß-cells from 12 week old mice (red) and genes that were increased >2 fold in ß-cells from 4 week old mice (blue). (**b**) Gene ontology analysis of GO terms arising from genes up-regulated >2 fold at 4 weeks of age. (**c**) Gene ontology analysis of GO terms arising from genes up-regulated >2 fold at 12 weeks of age. (**d**) IFN alpha and (**e**) IFN gamma induced gene transcripts highlighted on a volcano plots (p-value vs. fold change) depicting up-regulated (right quadrant) and down-regulated (left quadrant) gene expression in ß-cells isolated from 12 week old mice compared to 4 week old mice. P-Values were determined using a chi-square test.

As an illustration of the method’s utility, we explored how local inflammation during the development of type-1 diabetes impacted the gene-expression programs of ß-cells. In the NOD mouse, leukocyte infiltration of islets begins around the age of weaning and by 12 weeks insulitis is well established in all mice^12^. In our colony the spontaneous development of diabetes typically occurs between 12–20 weeks of age. To better understand how these cells are affected by the inflammatory environment we chose to compare the transcriptional profiles of ß-cells from normoglycemic 4 and 12 week old NOD mice. Microarray analysis revealed 82 genes up-regulated >2 fold and 52 genes down-regulated >2 fold in ß-cells from 12 week old mice compared to those from 4 week old mice (Fig. 5A, See Supplemental Table 1).

Initial examination of genes up-regulated in ß-cells at 4 weeks of age indicated a number of genes intricately linked to the cell cycle program. Unbiased analysis using the Gene Ontology (GO) database (http://www.geneontology.org/) implicated pathways involved in cell replication and division among the highest enriched pathways of the genes that were up-regulated at least 2 fold at 4 weeks of age (Fig. 5B). The total ß-cell mass expands during gestation and into the neonatal period, which subsides around the age of weaning^13^. These results are therefore likely indicative of the residual post-natal development program and representative of a subset of the genes important for the expansion of ß-cell mass during early life.

We also examined how insulitis impacts ß-cells. The insulitic lesion is highly inflammatory in nature; composed of various activated leukocytes, with soluble mediators which have the potential to alter gene expression in β-cells. Gene ontology analysis of genes up-regulated at least two fold at 12 weeks of age identified pathways implicated in immunological responses (Fig. 5C). Recent studies have identified interferon responses as a strong hallmark of type-1 diabetes development. In particular, type 1 interferon responses are detected locally in inflamed islets of NOD mice prior to disease onset^14^,^15^,^16^ and additionally in PBMCs from individuals at risk for development of type-1 diabetes^17^. Therefore we asked whether a similar interferon response could be observed directly from β-cells at the transcriptional level. Overlaying of type I and II Interferon gene signatures revealed a significant bias of these transcripts amongst genes up-regulated in ß-cells from 12 week old mice (Fig 5D,E).

## Discussion

The difficulty in identifying and isolating pure mouse ß-cell populations has hindered diabetes research. Current methods used for ß-cell isolation rely on either autofluorescence which can result in contamination with other endocrine cells, or anti-hormone antibodies which requires fixation and hinders most further biochemical or genomic explorations. We aimed to establish the use of a rapid pancreatic mouse ß-cell isolation method that could be completed quickly and yield viable and pure ß-cell populations for subsequent analysis. Our data show that i) mouse pancreatic ß-cells can be stained in whole islets, ii) that the flow cytometry sorted population is >99% pure and iii) that isolated cells can be used for subsequent studies.

Gene expression profiling studies of cytofluorometrically sorted mouse ß-cells provided new insights into the genetic programs during different ages and stages of type-1 diabetes development. Analysis of ß-cells from 4 week old NOD mice revealed the expression of genes involved in cellular proliferation. This finding was not entirely surprising and most likely represents the postnatal ß-cell mass expansion that subsides around the time of weaning. Most of these genes were not expressed or only expressed at lower levels in older ß-cells, which have a substantially lower rate of turnover^18^,^19^. However, this rather quiescent state of ß-cells in adult mice has been shown to be reversible under certain circumstances^20^ and is an area of very active investigation. Greater understanding of the specific genes and pathways involved in regulating the cell cycle of ß-cells and how they might be modulated is of particular interest to the development of therapies aimed at increasing ß-cell mass.

In the context of established insulitis, we identified a group of genes whose expression was amplified in response to local inflammation. Many of these genes are strongly associated with immunological response pathways, in particular with clear shifts in transcripts induced by both type-I and type-II interferons. How transcriptional changes in β-cells influence diabetes remains largely unclear. It is conceivable that while a subset of the genes induced by local inflammation may help protect β-cells from destruction, others may alternatively increase their susceptibility to death. Further investigation in this area is warranted and we believe that fluorescent markers of live β-cells such as the EP12-BTMR-X probe described will be of particular relevance in these types of studies.

Although we only reveal one, we envision multiple applications of this new isolation procedure including identifying proteins or pathways that could be targeted for reprogramming of other cells into ß-cells^21^. Furthermore, although we only evaluated the probe with mouse β-cells, the high affinity binding of the E4 peptide to GLP-1R is not species specific, which may allow the probe to be translatable to other species, making it a potential useful tool for many more areas of research.

## Methods

### Synthesis of fluorescent exendin-4 and fluorescent scrambled peptide

EP12-BODIPY TMR-X (EP12-BTMR-X) and the fluorescent scrambled peptide were synthesized in two steps as previously reported^8^. First, 11-azido-3,6,9-trioxaun-decan-1-amine (10 equiv.) was added to a stirring solution of BODIPY TMR-X NHS(10 mg/mL in DMSO) and reacted at RT for 5 mins. The resulting azide functionalized fluorophore was isolated by reverse phase high performance liquid chromatography (RP-HPLC) with a linear gradient (5–100%B over 10 mins; 2.5 mL/min; A: H_2_O w/ 0.1%TFA, B: MeCN) at a flow rate of 2.5 mL/min. Next, to a microwave vessel 90 μL (60 equiv.) of bathophenanthroline disulfonate (80 mM in water) and 113.1 μL (46 equiv.) of tetrakis(acetonitrile)copper(I) hexafluorophosphate (50 mM in MeCN) were added, followed by the previously isolated azide-functionalized BODIPY TMR-X (0.5 mg in 50 μl DMF) and (K12Pra)exendin-4 (HGEGTFTSDLSKQMEEEAVRLFIEWLKNGGPSSGAPPPS-NH2) or scrambled peptide (ESATLEAEVDTPraMIHGQLRLGPSSSGGFEWFEPNPGKPS-NH2) (1 mM in 1XPBS). The vessel was purged with argon and capped prior to microwave irradiation (60 °C, 30 W) for 5 min. The mixture was filtered through a 0.22 μm spin filter and isolated by RP-HPLC with a linear gradient (25–55% B 0.3–10 min 2.5 mL/min; A: H_2_O w/ 0.1%TFA B: MeCN). The final product was concentrated by rotary-evaporation to remove the organic solvents and redissolved in 1X PBS (10% yield, 25.6 uM, stability: 7 months at 4 °C). MALDI-ToF mass spectrometry was used to verify the mass of the product. A Nanodrop 1000 was used to determine yield. The binding affinity of the conjugate for GLP-1R was determined by a ^125^I-Exendin (9–39) receptor binding assay as previously reported^8^.

### Mice

Female C57BL/6J (000664) mice (6–10 weeks old) were used for flow cytometric and microscopy analyses experiments. Female NOD mice (4 and 12 week old) were used for microarray studies. All experimental procedures were performed in accordance with relevant guidelines and regulation and were approved by Harvard Medical School’s Institutional Animal Care and Use Committee.

### Pancreatic harvesting

Mouse pancreatic islets were isolated using a standard protocol. Briefly, pancreata were perfused via the common bile duct with 0.8 mg/mL Collagenase P (Roche) in HBSS. Excised pancreata were dissociated for 15 min at 37 °C. Islets were subsequently washed three times with HBSS and collected onto a 70 μm cell strainer. The cell stainer was inverted and rinsed into a dish with RPMI containing 10% FCS. Islets were then handpicked using a stereomicroscope.

### Islet cell sorting by flow cytometry

Isolated intact islets were incubated at 37 °C for 30 min with 50 nM EP12-BTMR-X and anti-CD45 APC (Biolegend) in RPMI supplemented with 10% FCS and then washed twice with PBS. Then, islets were dispersed with 0.25% trypsin for 10 min at 37 °C. Dispersed islet cell suspensions were washed three times, resuspended in 400 μl PBS with 2% FCS and filtered through a 70 μM strainer (BD). To exclude dead cells, DAPI was added immediately prior to sorting on a FACSAria. Sorted cells were gated as EP12-BTMR-X positive, CD45 negative.

### Assessment of ß-cell purity by flow cytometry

EP12-BTMR-X stained intact islets were dispersed with 0.25% trypsin for 10 min at 37 °C and fixed with 4% paraformaldehyde for 20 min and subsequently permeabilized with perm/wash buffer (BD). Intracellular staining was carried out in perm/wash buffer containing 2% rat serum with anti-insulin APC (R&D systems) and anti-glucagon (R&D Systems) conjugated to PerCP-Cy5.5 using the Lightning-link antibody labeling kit (Novus Biologicals) for 2 h at RT. Following extensive washing, cells were filtered and acquired on a LSRII cytometer (BD). Data was analyzed using FlowJo software (Tree Star).

### Immunohistochemistry

Immunofluorescence labeling of islets from female C57BL/6J was performed to visualize colocalization of the probe in insulin positive glucagon negative cells. The intact pancreas was incubated with EP12-BTMR-X (37 °C, 30 min, 50 nM) in RPMI supplemented with 10% FCS and then washed twice with cold PBS. The pancreas was embedment in OCT compound (Sakura Finetek) and flash-frozen in an isopentane bath on dry ice. The frozen tissues were sectioned (5-um thickness), mounted on microscope slides, and stored at –80 °C. Slides were then incubated at 4 °C o/n with primary antibodies diluted with 4% goat normal serum in PBS solution. The following primary antibodies were used for immunostaining: rabbit anti-insulin (1:25, Santa Cruz), rabbit anti-glucagon (1:1000, Millipore) rabbit anti-somatostatin (1:250, Dako) and rabbit anti-c-peptide (1:100, Cell Signaling). A species-matched secondary antibodies were used for immune detection (Alexa Fluor 488 conjugated goat anti-rabbit, 1:100, Life Technology). Nuclear counterstaining was performed using DAPI (Invitrogen). Immunofluorescence images were acquired with an epifluorescence microscope, BX63 (Olympus), with a Neo sCMOS Monochrome camera (ANDOR).

### Gene expression analysis

Islets were pooled from 3 NOD mice and (1–2 × 10^4^) EP12-BTMR-X positive cells were sorted per replicate (n = 2 per age group) into 500 μl of TRIzol (Invitrogen) for RNA isolation. ssDNA generated using the Ambion WT expression kit and Affymetrix WT GeneChip Terminal Labeling kit was hybridized to GeneChip Mouse Gene 1.0 ST arrays (Affymetrix). Raw data was normalized with the RMA algorithm and analyzed with GenePattern software. The cutoff level is based on values empirically determined and per standard operating procedures of the ImmGen protocol regarding microarray data generation and quality control. Specifically, genes with an expression level value of 120 or greater have a 95% probability of true expression, whereas genes with an expression level value of 60 or lower have a 95% probability of not being expressed and those with intermediate expression levels in the range 80–90 have a probability of 50% true expression. As a result of these observations, we routinely use a value of 100 as the cutoff level for detection for standard microarray analysis.

A type II interferon gene signature was obtained from a previous report^22^ and a type I interferon was generated from leukocyte populations stimulated *in vivo* with interferon-alpha (S. Mostafavi, in preparation). Microarray data are available from the National Center for Biotechnology Information/GEO repository under accession no. GSE64508.

## Additional Information

**How to cite this article**: Clardy, S. M. *et al.* Rapid, high efficiency isolation of pancreatic ß-cells. *Sci. Rep.* **5**, 13681; doi: 10.1038/srep13681 (2015).

## Supplementary Material

Supplementary Information
