# Supplementary material for: Rapid, high efficiency isolation of pancreatic ß-cells
Source: Sci Rep. 2015 Sep 2;5:13681. doi: 10.1038/srep13681 (PMC4557033; doi:10.1038/srep13681)
Supplement: Supplementary Information [file srep13681-s1.pdf]

Supplementary Information

Rapid, high efficiency isolation of pancreatic ß-cells

Susan M. Clardy[1], James F. Mohan[2], Claudio Vinegoni[1], Edmund J. Keliher[1], Yoshiko Iwamoto[1], Christophe Benoist[2,4], Diane Mathis[2,4], Ralph Weissleder[1,3,*]

[1]Center for Systems Biology, Massachusetts General Hospital, Harvard Medical School, Boston, Massachusetts

[2]Division of Immunology, Department of Microbiology and Immunobiology, Harvard Medical School, Boston, Massachusetts

[3]Department of Systems Biology, Harvard Medical School, Boston, Massachusetts, [4]Evergrande Center for Immunologic Diseases, Harvard Medical School and Brigham and Women's Hospital, Boston, Massachusetts*

Corresponding author, Dr. Ralph Weissleder

rweissleder@mgh.harvard.edu, phone: (617) 643-0500

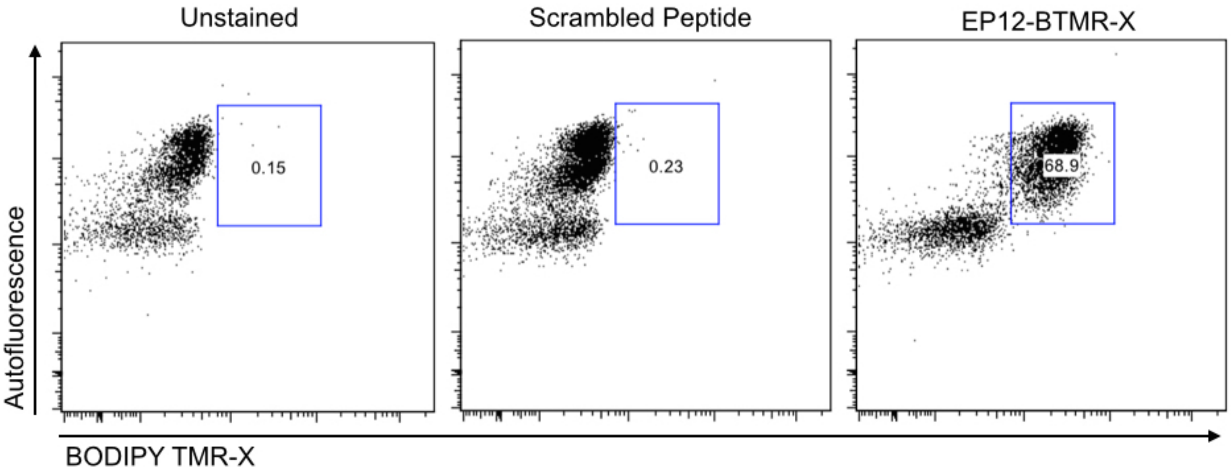

Figure S1. A dispersed mouse islet stained with BODIPY TMR-X labelled exendin-4 (EP12-BTMR-X) vs a labelled scrambled peptide. A side by side comparison of the fluorescent scrambled peptide vs EP12-BTMR-X performance in labelling mouse β-cells demonstrated the importance of the exendin-4 peptide sequence.

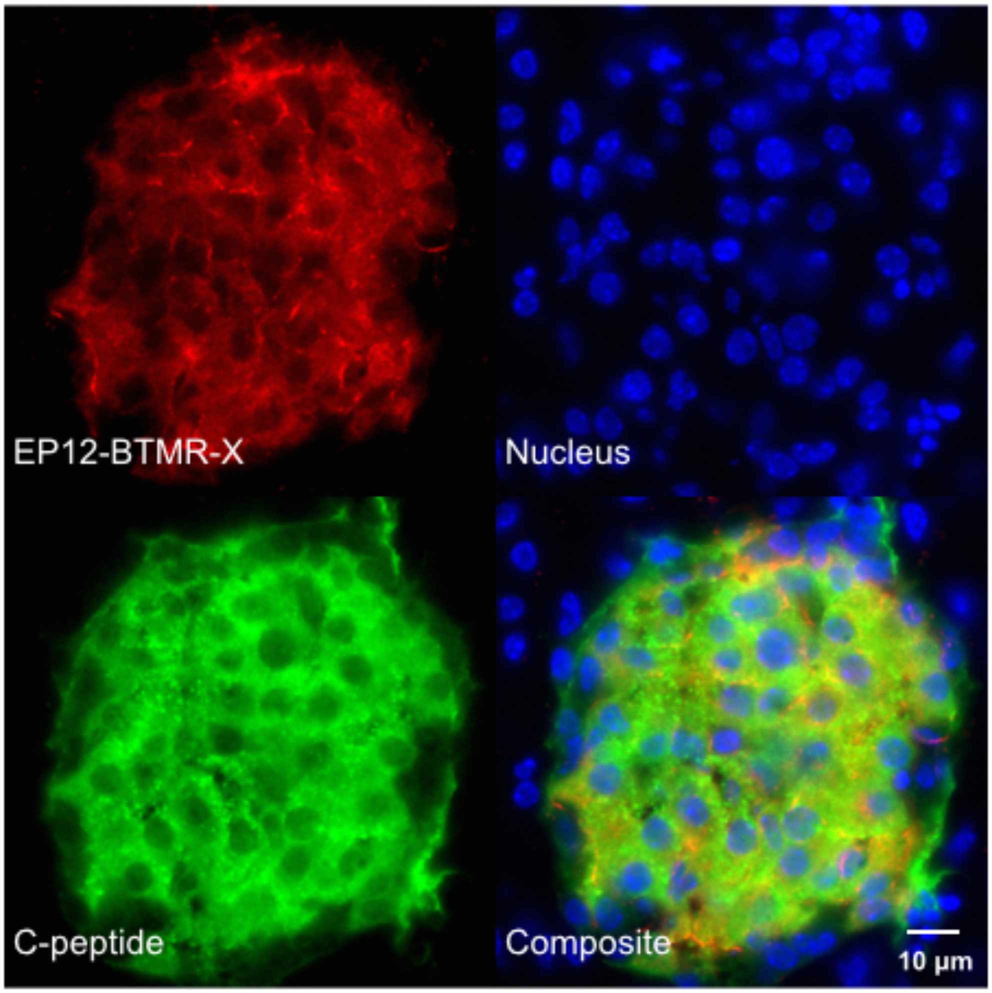

Figure S2. Immunostaining of ex vivo mouse pancreas for c-peptide. EP12-BTMR-X (red) accumulates in c-peptide positive (green) cells.

Table S1: Genes up-regulated >2 fold from 12 and 4 week old NOD mice.

Genes >2 Fold increased at 12 wks						Genes >2 Fold increased at 4 wks			
**GeneSymbol**	**Fold Change**	**GeneSymbol**	**Fold Change**	**GeneSymbol**	**Fold Change**	**GeneSymbol**	**Fold Change**	**GeneSymbol**	**Fold Change**
Hspa1a	9.3	Irgm1	3.1	Snora44	2.3	Msln	5.2	Hist1h2an	2.2
Cd74	8.9	Slfn3	2.9	Il18bp	2.3	Cdk1	4.5	Fads2	2.2
Cd274	6.2	H2-K1	2.8	H2-t9	2.3	Plk1	3.8	Padi2	2.2
Hspb1	6.2	Snora73b	2.8	Snora73a	2.3	Cks2	3.7	Hist1h2ad	2.2
Rnu12	6.0	Rgs2	2.7	Snhg1	2.3	Mki67	3.6	Hist1h2ah	2.2
Tgtp1	5.4	H2-Eb1	2.7	Rhob	2.2	Stmn1	3.6	Hist1h2ao	2.2
Gbp9	5.3	Fbp1	2.7	Parp14	2.2	Ube2c	3.1	Scarna17	2.2
Tnfsf10	4.7	Dbpht2	2.5	Serpinb9	2.2	Cel	3.0	Hist1h2ai	2.2
Gbp6	4.2	Serpina10	2.5	Mir344	2.2	Tcf19	2.9	Tubb5	2.1
Nupr1	4.2	Rprl2	2.5	Herc6	2.2	Cenpm	2.9	Zfp367	2.1
9930111J21Rik2	4.0	Rnu2	2.5	Gm12258	2.2	Ect2	2.9	Foxred2	2.1
Stat1	3.9	Tap1	2.5	Rtp4	2.2	Igf2bp2	2.9	Hist1h2ag	2.1
H2-Aa	3.9	Cd209a	2.5	Oas2	2.2	Asf1b	2.9	Scd1	2.1
Gbp3	3.9	Rnu3b1	2.5	Cttnbp2	2.2	Hist1h2ak	2.9	St8sia5	2.1
Cxcl10	3.9	Trp53inp1	2.5	Parp9	2.1	Pmp22	2.8	Mcm6	2.1
Taf1d	3.8	Srsf5	2.4	H2-Q8	2.1	Th	2.6	Mad2l1	2.1
H2-Q6	3.7	Vaultrc5	2.4	H2-D1	2.1	D2Ertd750e	2.5	Npnt	2.1
H2-Ab1	3.7	Serping1	2.4	Rgs4	2.1	Oat	2.4	Cisd1	2.1
BC023105	3.6	Eid1	2.4	H2-T23	2.1	Aurkb	2.4	Adora3	2.0
Snora3	3.6	B2m	2.4	Tmtc1	2.1	Ezh2	2.4	Otub2	2.0
Snora3	3.5	H2-Q7	2.4	Mir7b	2.1	Nt5dc2	2.3	Hist2h2ab	2.0
Rnu3a	3.5	Serpina3n	2.4	Oasl2	2.1	Nnat	2.3	5730403M16Rik	2.0
H2-gs10	3.4	Psmb9	2.4	Ube2l6	2.0	Hist1h2bb	2.3	Nuak1	2.0
Nlrc5	3.3	Gprasp2	2.3	Fam159b	2.0	Armcx2	2.3	Fam101b	2.0
Rprl1	3.3	Nmnat2	2.3	Ddx58	2.0	Cenpn	2.3	Vps37b	2.0
Aldh1l2	3.2	Sprr1a	2.3	Sat1	2.0	Elf4	2.3		
Tmem59l	3.1	Dnaja4	2.3	Hspd1	2.0	Rfc4	2.2		
